# Smart Nanomaterials for Biomedical Applications—A Review

**DOI:** 10.3390/nano11020396

**Published:** 2021-02-04

**Authors:** Magdalena Aflori

**Affiliations:** Petru Poni Institute of Macromolecular Chemistry, 41A Grigore Ghica Voda Alley, 700487 Iasi, Romania; maflori@icmpp.ro

**Keywords:** smart nanomaterials, stimuli-responsive polymers, biomedical applications

## Abstract

Recent advances in nanotechnology have forced the obtaining of new materials with multiple functionalities. Due to their reduced dimensions, nanomaterials exhibit outstanding physio-chemical functionalities: increased absorption and reactivity, higher surface area, molar extinction coefficients, tunable plasmonic properties, quantum effects, and magnetic and photo properties. However, in the biomedical field, it is still difficult to use tools made of nanomaterials for better therapeutics due to their limitations (including non-biocompatible, poor photostabilities, low targeting capacity, rapid renal clearance, side effects on other organs, insufficient cellular uptake, and small blood retention), so other types with controlled abilities must be developed, called “smart” nanomaterials. In this context, the modern scientific community developed a kind of nanomaterial which undergoes large reversible changes in its physical, chemical, or biological properties as a consequence of small environmental variations. This systematic mini-review is intended to provide an overview of the newest research on nanosized materials responding to various stimuli, including their up-to-date application in the biomedical field.

## 1. Introduction

From the oldest times, humanity has tried to mimic nature in the way that living organisms adapt their behavior to environmental conditions to improve survival. It is well known that living systems from nature can dynamically change their properties in a smart way for adapting to the surrounding environment. Some examples are given by the *Mimosa pudica* plant which responds to stimuli such as temperature and light by undergoing a change in leaf direction [1]; by pinecones [2], wheat awn [3], and orchid tree seedpods [4] which adopt different shapes, responding to the changing environmental humidity; and by the *Venus flytrap* [5] which is able to capture insects by rapidly closing its leaves, among many others [6,7]. The powerful abilities of the biological systems abovementioned in converting energy and executing multiple tasks inspired the researchers to develop “stimuli-responsive” materials with biomimetic behavior and a high potential of use in smart or intelligent devices.

To our knowledge, the first complete report of “intelligent materials” defined as “the materials that respond to environmental changes at the most optimum condition and manifest their own functions according to these changes” was made by Toshinori Takagi in April 1990 [8]. At that time, the coverage and the achievability of this concept was not comprehensible, but it was anticipated to open an unused field in science and innovation [9]. Nowadays, the term “intelligent material” is synonymous with “stimuli-responsive material” or “smart material” and has gained a growing interest in researchers’ concerns due to the development of advanced technologies and the increased need for new materials that meet the new requirements.

Richard Feynman, laureate of the Nobel Prize, was the first to introduce, in 1959, the nanotechnology concept. He had a revolutionary vision in a lecture entitled: “Why can’t we write the entire 24 volumes of the Encyclopedia Britannica on the head of a pin?” [10]. The term “nanotechnology” was used and defined in 1974, by Norio Taniguchi, as “nanotechnology mainly consists of the processing of separation, consolidation, and deformation of materials by one atom or one molecule” [11]. In 1997–1998, the perception of nanotechnology was more a “science fiction” vision, still being far from the practical applications [12], but in the early 2000s, nanomaterials were intensively studied and finally utilized in practice. As the field of biomedical engineering is developing new insights, the demand for highly functionalized biomaterials is increasing. Despite the astonishing diversity and complexity of living systems, they all share the possibility to react to environmental changes, crucial for maintaining normal functions. This need for adaptation has led to the development of smart nanomaterials, defined as materials that can react to a large variety of stimuli by adapting their own properties such as shape, surface area, size, permeability, solubility, mechanical properties, and others. Depending on the capacity of the nanomaterial to restore its initial state, the response can be reversible or not. In this specific situation, polymer-based materials have substantiated themselves as sharp choices in creating upgraded responsive frameworks on the grounds that their structure permits regulating their properties. A large variety of polymers have been obtained to respond to physical stimuli (temperature, light, ultrasound, electrical, magnetic, mechanical), chemical stimuli (pH, solvent, electrochemical), or biological stimuli (enzymes). A special type of polymer is dual or multi-stimuli-responsive, in light of the fact that it simultaneously reacts to multiple stimuli. As a rule, the polymer responsivity is directed by the science of the monomers and their distribution/concentration in the polymer chains [13].

Herein, a systematic overview of some of the newest and major advances in developing sustainable nanomaterials for biomedical applications is presented. This mini review exhaustively portrays newly developed strategies for preparing smart polymers and the potential and difficulties of assembling responsive systems utilized for imagistic or for therapeutic applications, such as smart drug delivery, tissue engineering, wound healing, theranostics, and so on. A special attention is dedicated to metallic nanoparticles for diagnosis and therapeutic applications, due to their highly tunable unique plasmonic properties [14]. We provide not only examples of how nanomaterials satisfy these requirements and the ways in which it is possible to emulate these characteristics in engineered nanoplatforms, but also the gaps that remain to be bridged.

## 2. Types of Stimuli

**Figure 1:**
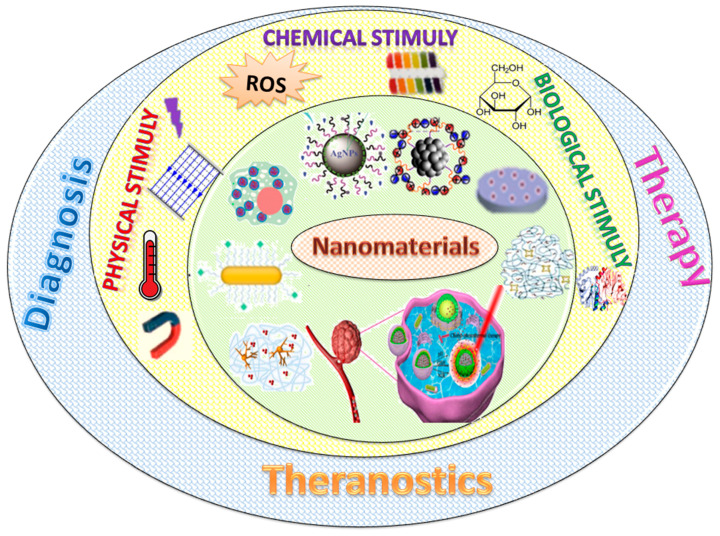
Classification and biomedical applications of smart nanomaterials as a function of their nanostructure.

Smart nanomaterials are categorized in different groups by means of the applied stimuli. The properties of smart nanomaterials are modified by external triggers in a controlled way [15,16,17,18,19,20,21,22,23]. By considering their various properties, many kinds of smart nanomaterials are known. These stimuli can typically be classified into three different categories: physical, chemical, and biological, as shown in Figure 1. The main applications in the biomedical field of these special materials as a function of their ability to respond to one or more stimuli are also highlighted in Figure 1.

### 2.1. Physical Responsive Nanomaterials

Examples of physico-sensitive nanomaterials and their applications are listed in Table 1.

#### 2.1.1. Temperature-Responsive Nanomaterials

Since 1942, when Huggins and Flory first theoretically described polymer–solvent interaction in solution with varying temperature and the concept of free volume (used to explain the critical temperature lower/upper phenomenon in solution), temperature-responsive polymers have received increasing interest in the biomedical field [21,22].

Thermosensitive polymers are a type of material that go through a sudden change in their solubility as a reply to a small temperature change [23,24,25], and they have stimulated researchers’ attention in the biomedical field, taking into consideration that specific infections show temperature changes [41]. Temperature-responsive polymers have a typical trademark highlight in the presence of a hydrophobic group: propyl, methyl, and ethyl groups. When warmed or cooled over a critical transition point, inside a small temperature range, a break of hydrophobic and intra/intermolecular electrostatic interactions takes place and the impact is a phase transition in the volume. The lower critical solution temperature (LCST) is the temperature above which the polymeric monophasic system endures phase separation and becomes biphasic, hydrophobic, and insoluble. On the contrary, below a lower critical solution temperature (LCST), the polymer is monophasic [26,42]. Above an upper critical solution temperature (UCST), one polymer phase appears, and below this, a phase separation exists [43]. In polymer solutions, the LCST usually results from a coil to a globule transition, minimizing the contact with the solvent [44]. The UCST is normally present in soluble polymers [45,46]. A particular class of temperature-responsive materials are the polymers that can display both LCST and UCST properties, but at different temperatures [47,48]. For better results, the LCST value of a stimuli-responsive system must be close to the body temperature and by increasing that value to approximately 42 °C, due to a change in the environmental condition, the initially loaded drug is released. Notwithstanding the LCST, the engineering of nanomaterials might be utilized to adjust drug release kinetics, especially with huge changes in the surface area as a result of the modified porosity and geometries or inclusion of metallic nanoparticles. Some parameters such as polymer concentration and molecular weight or pH can impact the LCST and UCST behaviors of thermoresponsive polymers [49,50,51].

In a recent study, Tamaki et al. obtained thermoresponsive polymers by different phenylalanine (Phe)-modified zwitterionic dendrimers [50]. They obtained carboxy-terminal Phe-modified dendrimers polyamidoamine (PAMAM) and succinic anhydride (Suc) (PAMAM-Suc-Phe and PAMAM-Phe-Suc) utilizing PAMAM dendrimers modified with Phe and Suc. Both these dendrimers indicated UCST-type thermosensitivity. Strangely, PAMAM-Phe-Suc showed LCST-type thermosensitivity at lower pH, but PAMAM-Suc-Phe did not. This shows that, as a function of the pH solution, PAMAM-Phe-Suc can switch LCST/UCST-type thermoresponsivity and the inner tertiary amine and the Phe residue in the dendrimer are indispensable for their stimuli-sensitive behaviors. These dendrimers induced coacervation (liquid–liquid phase separation) during temperature changes. Tissue engineering, drug delivery, or diagnosis applications from thermoresponsive nanomaterials were achieved from different hydrogels with various nanostructures and behaviors [27,28,52,53,54] or smart polymer substrates [55]. However, for the as-mentioned biomedical applications, responsive behavior must be offset with biocompatibility and degradation kinetics [53].

**Figure 2:**
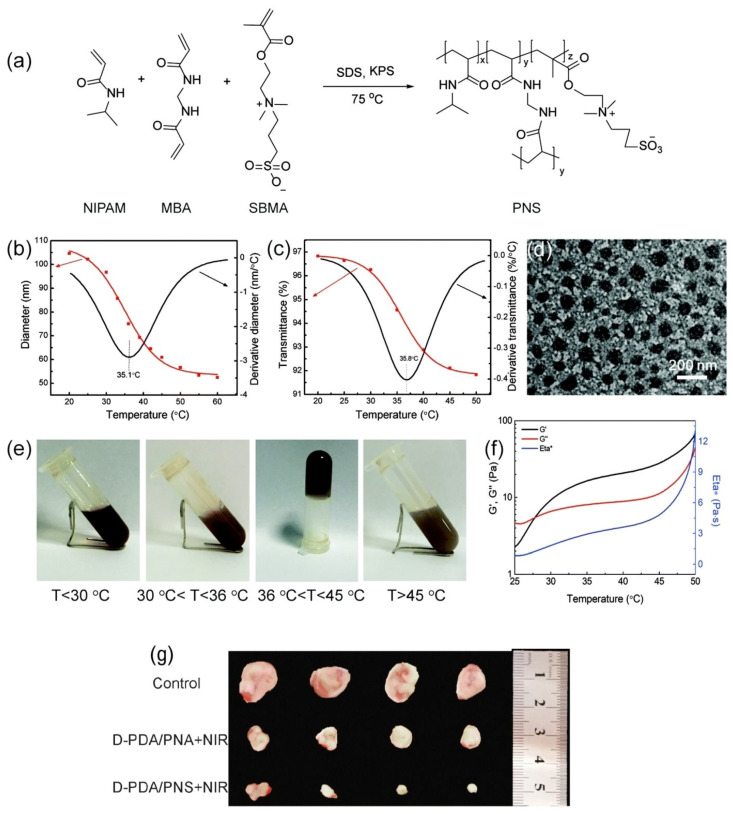
Zwitterionic thermosensitive PNS nanogels. (**a**) Schematic synthesis of PNS nanogels. (**b**) Temperature dependence of hydrodynamic diameters and (**c**) temperature dependence of PNS transmittance. (**d**) TEM image. (**e**) Photos of the sol–gel phase transition. (**f**) Rheology of the sol–gel phase transition. (**g**) *In vivo* antitumor effect: the corresponding photographs of the peeled tumors after intratumoral injection for 14 days. Reproduced from [29], with permission from © The Royal Society of Chemistry 2020.

Zheng et al. developed an injectable low-fouling zwitterionic thermosensitive hydrogel [29] (Figure 2). The zwitterionic thermosensitive poly(Nisopropylacrylamide-co-sulfobetaine methacrylate) (PNS) nanogels were synthetized using N-isopropylacrylamide (PNIPAM), sulfobetaine methacrylate (SBMA), and N, N’-methylenebisacrylamide (MBA). The average hydrodynamic diameter of nanogels was ca. 105 nm (Figure 2b). The sol–gel phase transition of the nanogel is based on the balance between the hydrophobic interaction between isopropyl groups in PNIPAM segments and the hydrogen bonding between the amide groups in poly(N-isopropylacrylamide) PNIPAM chains and water (Figure 2c). Under around 30 °C, hydrogen bonding was predominant and the fluids were transparent. After the temperature was expanded to 36 °C, a shrank gel was formed by the sol–gel phase transition because of further improvement in the hydrophobic interaction between isopropyl groups. In this cycle, the water was extracted from the nanogels, prompting phase separation of the formed gel.

A recent study involving thermosensitive liposomes was developed for drug delivery applications [56]. The authors obtained a nanosystem engineered by encapsulation of indocyanine green (ICG) and L-buthionine sulfoximine (BSO) into NIR photothermal liposomal nanoantagonists (PLNA) for amplified photodynamic therapy, through reducing intracellular glutathione (GSH) biosynthesis.

#### 2.1.2. Electrical and Electrochemical Stimuli-Responsive Nanomaterials

Electroresponsive materials are materials which adjust their physical properties (size or shape) as response to a small change in the applied electric current. Electroresponsive polymers possess a generally huge number of ionizable groups and are capable of transducing electrical energy into mechanical work.

The main applications of this type of smart material are artificial targeted drug release, muscle actuations, and energy transductions. These materials present the advantages of exact control through the span of an electrical pulse, the current intensity, or the time between pulses. A disturbance in the hydrogen bonding of polymer chains because of the applied electric current generates an adjustment in pH which induces polymer chain degradation or bending and, finally, drug delivery. Significant mechanisms engaged in drug delivery from electrosensitive polymers are charged drug electrophoresis, diffusion, and drug release after erosion of electro-erodible polymers [30,57,58]. Two classes of electroresponsive materials are known. The first class is formed by current-responsive polymer materials [31,59] in which an adjustment in the ions’ local concentration in materials or solution is due to the ions’ mobility induced by the electric field (example: hydrogels conductive polymers, layer by layer coatings) [60,61,62]. The second class is formed by voltage-responsive polymers predominantly utilized in biomedical applications (dielectric gels, elastomers, polymers for controlled drug release, accumulation on electroresponsive nanoparticle drug release, etc.) [63]. Gel bending because of an applied electric field relies on various factors, for example, the applied voltage, the thickness or shape of the gel, the position of the gel relative to the electrodes, and variable osmotic pressure. Electrosensitive materials can be obtained from natural polymers (alginate, hyaluronic acid, and chitosan) or synthetic polymers (acrylonitrile, allyl amine, methacrylic acid, vinylacrylic acid, and vinyl alcohol). Sometimes, blends between synthetic and natural polymers have been proven as viable possibilities for electroresponsive systems. Polymers such as polyaniline (PAni), polypyrrole (PPy), and polythiophenes (PTh) are electroresponsive materials often used in drug delivery applications [61]. Polyelectrolytes are a type of polymer that possess electrosensitive behavior, and, after application of an electric field, they suffer deformation due to anisotropic swelling or deswelling as the charged ions move towards the anode or cathode. The gel deformation is driven by the stress gradient (at the cathode, a smaller stress, and near the anode, the greatest stress).

Electrically conductive polymers could be engineered for devices such as biosensors, substrates for neural prostheses, and drug release platforms [32,33,34,35]. For example, Abidian et al. obtained the sustained release of individual drugs and bioactive molecules for designing low-impedance, biologically active polymer coatings, for better integration of electronically active platforms with living tissues. In their study, the release of dexamethasone was precisely controlled by external electrical stimulation of poly(3,4-ethylenedioxythiophene) nanotubes. They produced nanofibers of biodegradable poly(L-lactide) or poly(lactide-co-glycolide) onto the surface of a neural probe and coated with conducting polymers. In a last advance, the fiber layouts permitted gradual degradation, providing sustained release of biologically active agents [30].

**Figure 3:**
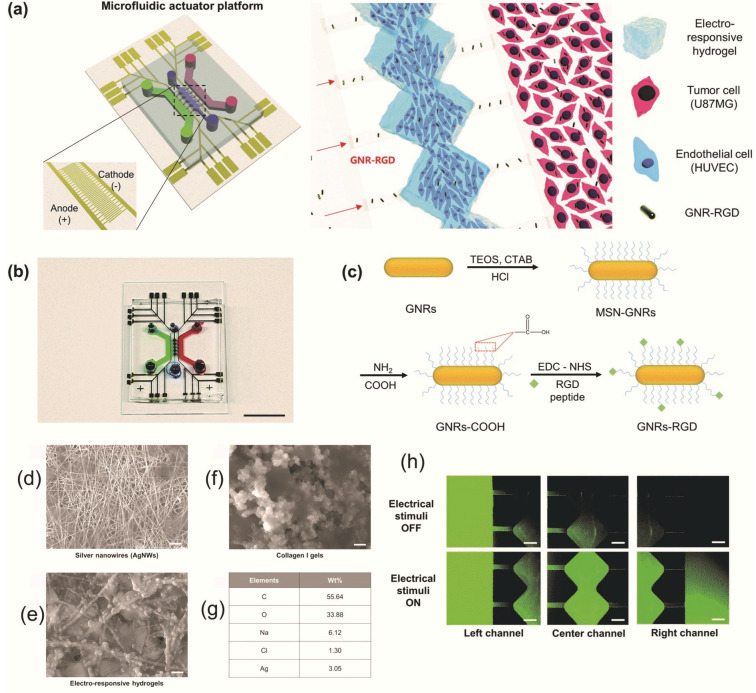
The hydrogel-based electroresponsive microfluidic actuator platform: (**a**) Illustration of the components of the electroresponsive microfluidic actuator platform. (**b**) Photo of microfluidic system, scale is 1 cm. (**c**) Proposed synthesis of peptide-conjugated gold nanorods. SEM images of (**d**) silver nanowires, (**e**) collagen I gels, and (**f**) electrosensitive hydrogels. (**g**) Elemental analysis of the electrosensitive hydrogel, scale is 1 µm. (**h**) Fluorescent images of “on/off” electrical stimuli influence on the fluorescein isothiocyanate–dextran propagation in the platform, scale is 500 µm. Reproduced from [57], with permission from © The Royal Society of Chemistry 2020.

Ha et al. built a microfluidic actuator platform based on an electroresponsive hydrogel used in photothermal therapy (PTT) and brain tumor targeting applications. The hydrogels were obtained from collagen I gel and silver nanowires (AgNWs) with high conductivity (Figure 3) and responded to electrical stimuli. In addition, they effectively exhibited PTT adequacy for brain tumors using arginylglycylaspartic acid peptide-conjugated gold nanorods [57].

An important type of electrical-responsive system is represented by electrochemical biosensors, due to their numerous points of interest, such as effortless utilization and sustained tracking at a fair cost benefit. These highlights make electrochemical biosensors supportive instruments in DNA mutation and cancer marker discovery in clinical tests, such as the diagnosis of acute lymphoblastic leukemia. In this context, a DNA electrochemical biosensor containing poly(catechol), graphene sheets, and gold nanoparticles was produced [35].

#### 2.1.3. Light-Responsive Nanomaterials

**Figure 4:**
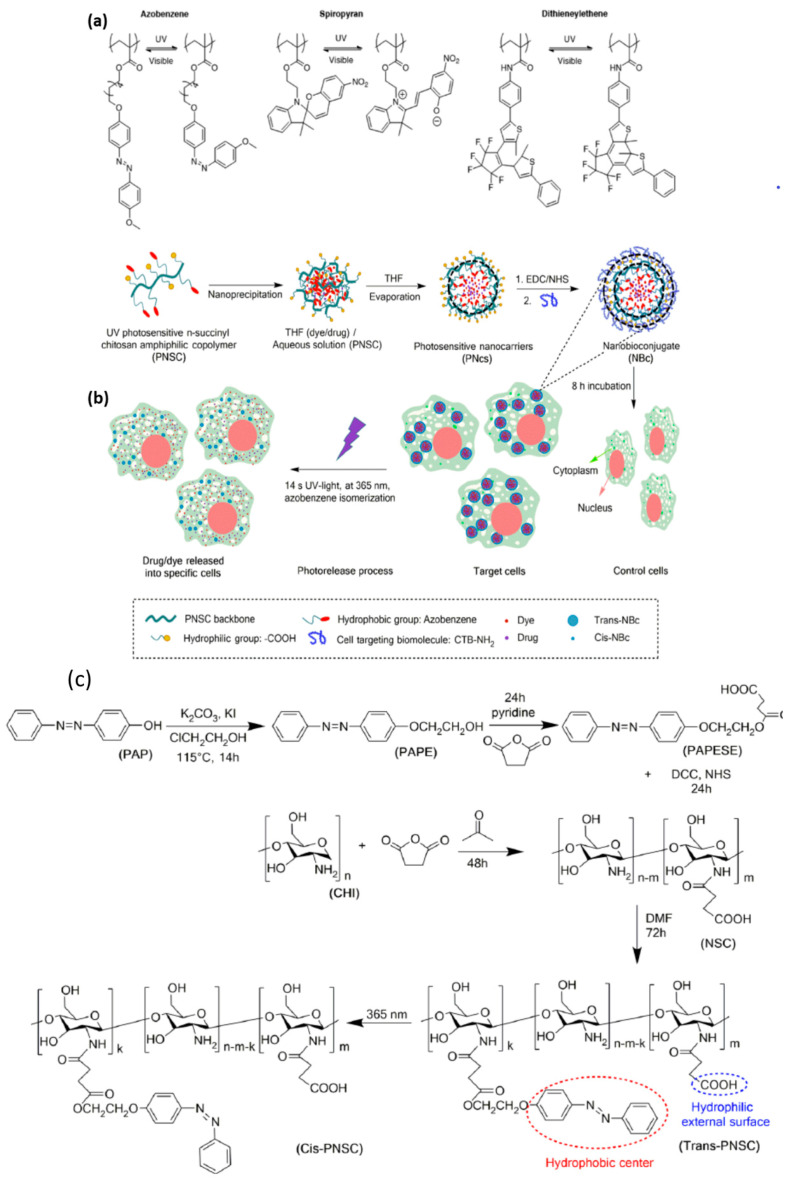
(**a**) Examples of photoisomerizable groups for reversible light-responsive nanomaterials. Reproduced from [65], with permission from © The Royal Society of Chemistry 2017. (**b**) Synthesis of PNc and UV light drug release. (**c**) Synthesis and photoisomerization process of PNSC amphiphilic copolymer. Reproduced from [70], with permission from © 2020 Mena-Giraldo, Perez-Buitrago, Londono-Berrío, Ortiz-Trujillo, Hoyos-Palacio, and Orozco under CC BY 4.0.

Light is regarded as an appealing stimulus because of its controllable and tunable properties. Light-responsive materials are profoundly invaluable for applications since light can be applied immediately and with extremely high spatiotemporal accuracy with an on/off controlling mode. Moreover, light-sensitive intelligent materials are biomarkers which indicate the site of drugs and the targeting capacity and visualize the tumors by fitting a wide variety of parameters. Considering specific applications, an advantage of this stimulus material is that input parameters such as intensity, wavelength, light, beam diameter, and exposure time can be easily tailored. To overcome the issues related to the precise drug release or light-mediated theranostics, smart nanomaterials such as nanopolymers are an alternative way in which the drug delivery on target locations can be controlled and the right concentration of drug delivery in a specific time can be achieved. Several light-sensitive polymers contain chromophores: azobenzene groups [64], spiropyran groups [36], or nitrobenzyl groups (Figure 4a) [65]. Various photomaterials were effectively utilized for biomedical applications, but unfortunately the greater part of them is activated only in the UV/VIS region, which restricts tissue penetration and destructive behavior related to ordinary tissues. The development of photoresponsive smart nanomaterials is an approach to overcome these difficulties on the grounds that these materials can successfully create reactive oxygen species (singlet oxygen, peroxide, hydroxyl, etc.) and have targeting ability, good solubility in water, and near-infrared light activation (NIR) [37].

Photoabsorbing materials are widely used in photothermal therapy due to local hyperthermia which kills cancer/bacteria cells. In biological media, an environment that is overheated produces few risky impacts: cell lysis, protein evaporation of the cytosol, and aggregation or denaturation [66]. As referenced before, the majority of the recent phototherapeutic systems adequately use NIR-sensitive nanopolymers to defeat the downsides related to photosensitizers, similar to UV/VIS-sensitive fluorophores [67]. Furthermore, most of the recent diagnosis or therapeutic systems are using NIR fluorescence for observing deep tissue cancer tumors [68]. Mimicking natural structures, the production of photoresponsive polymers can be achieved by introducing photoactive molecules in the side chains or the polymeric backbone by exposition to UV or near-infrared (NIR) irradiation. Those materials undergo conformational, stereochemical, or structural changes, such as photoisomerization and photocleavage. For azobenzene, this process is visualized by the change in molecular symmetry from a thermally stable *trans* (E) orientation to a less favorable *cis* (Z) orientation. In spiropyrans, a ring-opening reaction is induced by the irradiation with the appearance of the isomeric merocyanine form. By UV absorption, the photoactive groups from the nanocarriers suffer a reversible isomerization from *cis* to *trans* and the trans isomer can be reconverted into the *cis* isomer by visible light. In this way, the nanostructures are disrupted and their cargo is released [69].

Mena-Giraldo et al. synthesized a photoresponsive polymeric nanocarrier by chitosan modification with ultraviolet-photosensitive azobenzene molecules (Figure 4b). They considered the impact of UV light on releasing the cargo in a guided manner by means of nanobioconjugates. As cargo models, they chose Nile red and dofetilide and proved the encapsulation/release model. They used photoresponsive polymeric nanocarriers functionalized with a cardiac transmembrane peptide. The viability of the therapeutic regime was improved by the effect of UV light irradiation which increases the concentration of intracellular delivery and decreases the amount and cargo reactions [70]. In Figure 4, the notations are: 2-(4-phenylazophenoxy)ethanol (PAPE); 2-(4-(phenylazo)phenoxy)ethanoloxy succinyl ester (PAPESE); N-succinyl chitosan (NSC); N-succinyl-N-4-(2-(4-(phenylazo)phenoxy)ethanoloxy)-succinyl-chitosan (PNSC); and chitosan (CHI).

A group of authors anchored uniform zeolitic imidazolate framework (ZIF-8) crystals onto FeOOH nanorods through a simple recombination process to obtain FeOOH@ZIF-8 composites for *in vitro* direct metabolic analysis of biofluids in diagnostics of gynecological cancers. The obtained FeOOH@ZIF-8 nanorods displayed an enhanced ionization efficiency and size-exclusion effect and had strong absorption at a wavelength of 355 nm, transferring photon energies absorbed from the UV laser light to analytes for fast, sensitive, and selective LDI-MS analysis of small metabolites in serum (circa 1 mL) without any pretreatment. The authors performed a photoelectrochemical analysis to provide insight into charge separation at the interface of FeOOH@ZIF-8 nanorods and they noticed that the photocurrent response of nanorods is enhanced by ZIF-8, suggesting that ZIF-8 decoration would reduce the rate of electron–hole recombination in FeOOH [71].

#### 2.1.4. Magnetic-Responsive Nanomaterials

The stimulus agent for magnetoresponsive nanomaterials is an applied magnetic field.

Particularly, magnetic nanoparticles are very useful in obtaining improved devices for theranostics. More specifically, those materials can improve magnetic separation, magnetic hyperthermia treatments, cellular labeling, immunoassays, magnetic-guided drugs, and magnetic resonance imaging diagnosis (MRI). Various techniques were employed for the synthesis of polymeric magnetoresponsive nanomaterials such as hydrothermal process, microbial synthesis, the co-precipitation method, combustion, laser pyrolysis, chemical vapor deposition, carbon arc, high-temperature thermal decomposition, and electrochemical synthesis. Magnetic nanoparticles such as iron, cobalt, nickel, and metal oxides have an important advantage for *in vitro* and *in vivo* remote control of cells, facilitating the understanding of cell functions and signaling. The traditional methods such as magnetic hyperthermia (using ultrasounds or microwaves, an increase in temperature above 45 °C in a small region or in the whole body results in killing cancer cells by thermotherapy) or thermal ablation (the diseased area is heated at above 45 °C) have some disadvantages such as low targeting ability and deep tissue penetration [72]. MRI was developed by Paul Lauterbur in 1973 and has become widely used around the world’s hospitals after it received Food and Drug Administration approval for clinical use in 1985 [73]. The MRI principle is based on the alignment of protons under an applied external magnetic field. A special class of pharmaceuticals is MRI contrast substances which increase the relaxation rates of water protons in target tissue and improve the image contrast [74]. The most used MRI contrast agents are based on clinical gadolinium [75]. Nowadays, improving the current MRI methods is required to overcome some disadvantages such as toxicity and sensitivity in some contrast media or delayed imaging rate and accuracy [38]. Therefore, various contrast agents based on iron nanoparticle systems were created to additionally enhance the signal to background noise ratio [39,76]. The magnetoresponsive nanomaterials of the future must be biocompatible with high contrast capacity and must be stable (the image quality is influenced by the degradation of the contrast agents).

**Figure 5:**
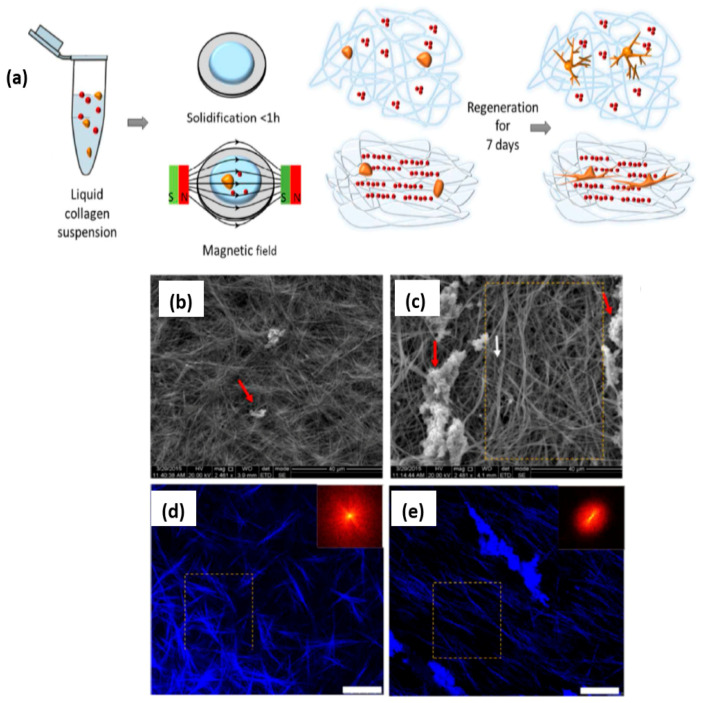
Neuronal regeneration in a magnetic-responsive 3D hydrogel system. (**a**) Schematic presentation of magnetic-driven neuronal regeneration. SEM images of (**b**) random collagen fiber orientation from spontaneous solidified suspension; (**c**) aligned collagen fiber from magnetic field-directed solidified suspension. Confocal reflectance microscopy images of (**d**) random collagen fiber orientation from spontaneous solidified suspension; (**e**) aligned collagen fiber (light blue) from magnetic field-directed solidified suspension. Reproduced from [40], with permission from © 2016 American Chemical Society.

An example of magnetoresponsive nanomaterials application is the system developed by Antman-Passig et al. (Figure 5) [40]. They obtained the alignment of collagen fibers prompted by the presence of magnetic nanoparticles (MNP) (red). The solidification of the collagen suspension containing neurons (orange) was observed both naturally (up) and under an applied magnetic field (red-green bars) (down). The first gel (up) presented an arbitrary collagen fiber direction (blue lines) and irregular distribution of MNP (red). The second gel (down) presented aligned collagen fibers (blue lines) and an aggregation of MNPs (red particles), while the neuronal development after a week produced neuritis, demonstrating the capacity of the system for 3D neural regeneration directed by a magnetic field.

### 2.2. Chemical-Responsive Nanomaterials

Examples of chemical-sensitive nanomaterials and their applications are listed in Table 2.

#### 2.2.1. pH-Responsive Nanomaterials

pH stimulus smart nanomaterials react to the pH by displaying new functional properties and are fascinating in the biomedical field because pH changes are present in many specific or pathological systems. The advantage of using those materials is that various segments of the human body have diverse pH levels (for chronic wounds, pH values are 7.4–5.4; for saliva 6.5–7.5; along the gastrointestinal tract the pH changes from the stomach (4–6.5) to the intestine (5–8)) [89]. Additionally, the pathological state shows strange pH values contrasted with the physiological state pH, for example, tumor micromedia have a lower extracellular pH between 6.5 and 6.9, bacterial infections present acidic pus with pH in the range 6.0–6.6, and an inflamed tissue has a pH value of 6–7.

In the light of these extraordinary pH varieties, different pH-responsive materials have been obtained so far. The pH-sensitive polymers are mostly classified as polymers with ionizable moieties and polymers with acid-labile linkages. The critical component for the first category is the existence of ionizable, fragile basic, or acidic moieties (amines and carboxylic acids) that bind to a hydrophobic backbone, for example, polyelectrolytes [90]. A common pH-responsive material of this class of polymer displays protonation/deprotonation processes by dispersing the charge over the ionizable groups of the molecule. The second class is the polymers with a backbone which involves acid-labile covalent linkages. A dissociation of polymer aggregates or a breaking in polymer chains is determined by the cleavage of these bonds at the reduction in pH. The second category has a slower inner alteration due to the presence of covalent bonding, which encourages their implementation in the drug release field. pH initiates a phase sudden transition in pH-responsive polymers. Ordinarily, the phase switches within a pH range of 0.2–0.3. The most known pH-sensitive polymers are poly(L-lysine), poly(N,N -dimethylaminoethyl methacrylate), poly(methacrylic acid), poly(acrylic acid), poly(N,N -dialkyl aminoethyl methacrylates), poly(ethylenimine), chitosan, aginate, and hyaluronic acid [91].

Experiments for *in vivo* wound healing demonstrated that pH-responsive silver nanoparticle clusters formed from the ortho ester inner layer and PEG corona (AgNCs) are effective for the healing of methicillin-resistant *Staphylococcus aureus* infections [77]. Transistors are usually utilized as electronic switches since they react pointedly with an amplified current output to an applied voltage threshold. A new hybrid nanoparticle design with a combination of three pH-responsive block copolymers, each of them acting as a sensor at a foreordained pH value, was reported to study endocytic organelles acidification from living cells. This ultra-pH-sensitive (HyUPS) nanotransistor with pH transitions at 6.9, 6.2, and 5.3 opens new insights in biomedical applications [78].

A novel pH-responsive supramolecular nanomaterial was developed, taking into account the negatively charged octasulfonate-modified zinc (II) phthalocyanine (ZnPcS8) which interacts with positively charged hydroxide double layers (LDH). In neutral conditions, LDH-ZnPcS_8_ is not photoactive, being activated in an acidic environment (pH 6.5) [79]. A melanin-like nanoparticle (MelNP) was obtained to exploit its unique characteristic in developing a highly pH-sensitive tool for *in vivo* cancer target imaging [80].

As a confirmation of the idea, a few pH-sensitive nanomaterials are obtained either from polymers with weak acid bonds (vinyl ester, ketal, acetal, orthoester, etc.) whose breaking initiates the surface charge changes or the molecules release, or from polysaccharides (e.g., chitosan) that suffer conformational changes and/or pH-responsive solubility. For instance, chitosan is a polysaccharide with different behavior: under neutral conditions, which are found in a healthy environment, it has a negative or neutral surface charge, while under an acidic condition from a pathological environment, it has a positive surface charge (due to the amino group’s protonation), which easily interacts with the negative surface charge of the cells. This behavior entitles chitosan to be utilized for targeting and giving specific sites for therapeutic viability [92].

Bonadies et al. developed a pH stimuli-responsive polylactic acid PLA system acting as an implant coating for prolonged period utilization. They described a Resveratrol (RSV)-containing membrane obtained by the electrospinning method, which liberated RSV very fast if pH decreased from a neutral to slightly acidic value around 5.5 (the case of infections). They demonstrated the PLA-RSV membrane’s capability to prevent implant-associated infections [81].

**Figure 6:**
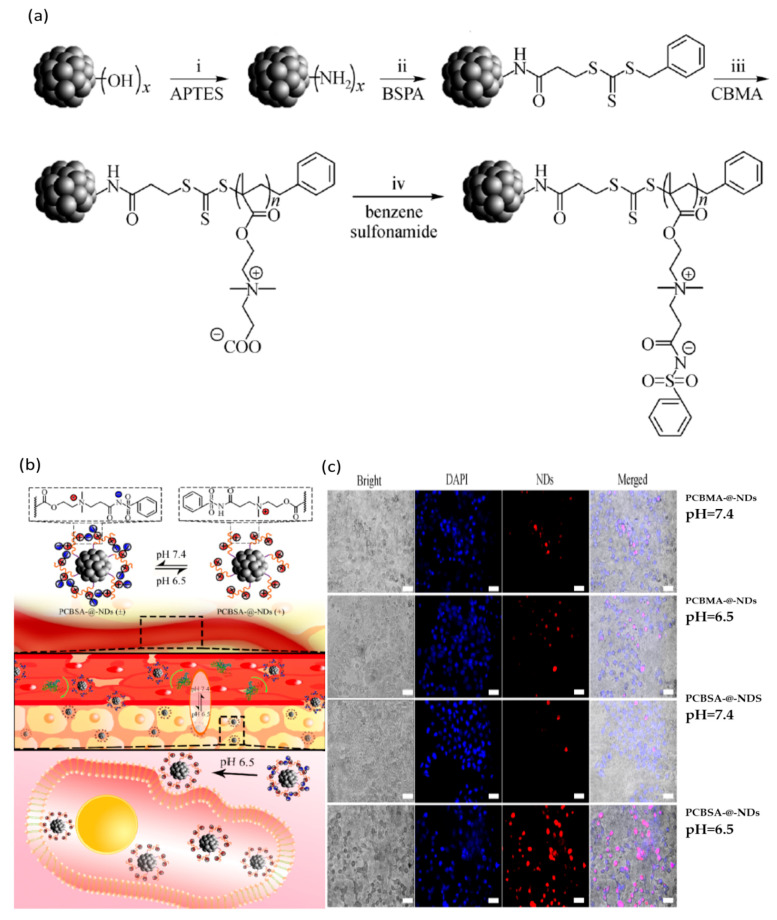
(**a**) Illustration of synthesis of PCBSA-@-nanodiamonds (NDs): 3-Amin-opropyltriethoxysilane (APTES) (i); 3(((benzylthio)carbonothioyl)thio)propanoic acid (BSPA) (ii); carboxybetaine methacrylate (CBMA) (iii); benzene sulfonamide (iv). (**b**) Schematic representation of the PCBSA-@-NDs’ performance at surface conversional charge and tumor cell uptake; (**c**) images of fluorescence microscopy for HepG2 treated with PCBMA-@-NDs at different pH values (scale is 50 µm). Reproduced from [82], with permission from © Higher Education Press 2020.

An illustration of a pH-responsive nanomaterial obtained by Zhou et al. is represented by a novel polyzwitterion coating nanodiamonds (ND) system designed to improve the fluorescence intensity and enhance antifouling properties, for *in vivo* imaging applications (Figure 6). They used poly(carboxybetaine methacrylate) PCBMA grafted on NDs (PCBMA-@-NDs) (Figure 6a) modified with benzene sulfonamide (PCBSA-@-NDs) for obtaining pH responsiveness. Fluorescence microscopy (Figure 6b) proved to offer superior imaging and better cell affinity for PCBSA-@-NDs as compared to PCBSA-@-NDs at tumor, slightly acidic pH [82].

#### 2.2.2. Redox-Responsive Nanomaterials

The redox-responsive nanomaterials have emerged as efficient biomaterials and, in particular, the nanoparticles have been broadly researched as efficient carriers for drugs, genes, and antigens in the forms of dendrimers, nanogels, and polymeric micelles [93,94,95]. Polymers containing labile groups are a good choice for developing redox-responsive biomedical systems. Typical redox-responsive polymers are poly(b-amino esters), polyanhydrides, and poly(lactic/glycolic acid) because they contain acid-labile moieties [83,84,85,96,97]. Most common redox-responsive agents for controlled drug release applications are thiol groups, platin conjugation, and thioether, disulfide, or diselenide bonds. Polymers with disulfide groups degrade in a reducing media such as cysteine or GSH [98,99,100].

The redox-active tripeptide γ-l-glutamyl-l-cysteinyl-glycine, or GSH, is an antioxidant synthesized in cells, being used to repress reactive oxidative species (ROS) agglomeration in sick tissues. The concentration of glutathione in tumoral tissues is 100 times greater than that in healthy tissues. Since GSH possesses thiol groups which participate in cleavage of disulfide groups, it represents a powerful reducing agent which prevents the accumulation of reactive oxidative species (ROS) in inflamed tissues and is very effective in anticancer drug delivery. A study demonstrated that the ROS concentration is 10–100 times more in colon cancer diseased tissue than in healthy tissue [101]. Polymers which possess a disulfide linkage and are very effective in micelles formation enable a response to GSH by *in vivo* micelle disruption to release the drug [102]. Different ROS-receptive DDS structures, including thioketal, thioether, aminoacrylates, polysaccharide, and polyproline, have been investigated in drug delivery systems. In an ongoing report, PEG_2000_-S-S-PTX micelles were made and portrayed for use in breast cancer applications, as redox-responsive prodrugs [86]. Other authors demonstrated the controllable anticancer capacity of selenium-based materials used as prodrugs and as anticancer drug vehicles. [87].

**Figure 7:**
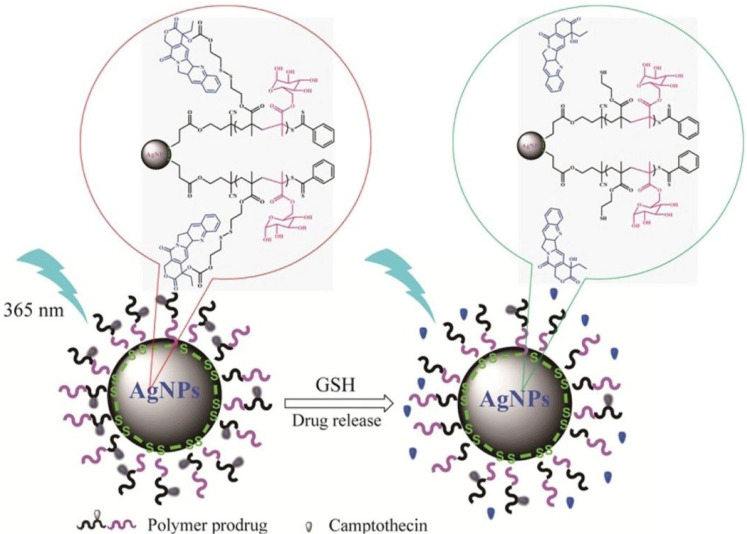
Representation of fluorescence “off” and “on” with the release of CPT from redox-responsive P(MACPTS-co-MAGP)@AgNPs nanoparticles. Reproduced from [88], with permission from © 2020 Elsevier.

Recent work described a hybrid nanoparticle-based system for controlled release of anticancer drugs. The system was obtained from P[(2-((2- ((camptothecin)-oxy)ethyl)disulfanyl)ethylmethacrylate)-co-(2-(D-galactose)methylmethacryl-ate)] P(MACPTS-co-MAGP)@AgNPs nanoparticles and camptothecin (CPT) was linked to the system by disulfide bonds. As shown in Figure 7, in the presence of GSH, the release of the drug is initiated, leading to the fluorescence “turn-on” of CPT [88].

### 2.3. Biological-Responsive Nanomaterials

Examples of biological-sensitive nanomaterials and their applications are listed in Table 3.

#### 2.3.1. Glucose-Responsive Nanomaterials

Sugar-sensitive nanomaterials can mimic normal endogenous insulin production as a response to the presence of glucose by limiting diabetic disorders and by delivering the bioactive agent in a driven way. In particular, polymers have gathered extensive consideration in view of their use in the glucose detection and insulin delivery fields. Despite these points of interest, the significant disadvantages to be overcome are the short reaction time and the low biocompatibility [114].

Glucose-responsive polymeric systems are precisely engineered to produce insulin and are based on enzymatic oxidation of glucose by glucose oxidase (GOx) and binding of glucose with lectin. A pH change in the environment is caused by glucose oxidation to gluconic acid. The response to the pH change is a volume transition of the polymer, and in this manner, the body’s glucose level is driven by conformational polymer changes [115]. Some authors synthesized acetalated dextran nanoparticles or acryloyl cross-linked dextran dialdehyde (ACDD) nanocarriers containing enzymes and insulin to facilitate glucose-sensitive release [103,116]. Boronic acid-derived hydrogels were used to obtain glucose-sensitive platforms for drug delivery [104,117].

**Figure 8:**
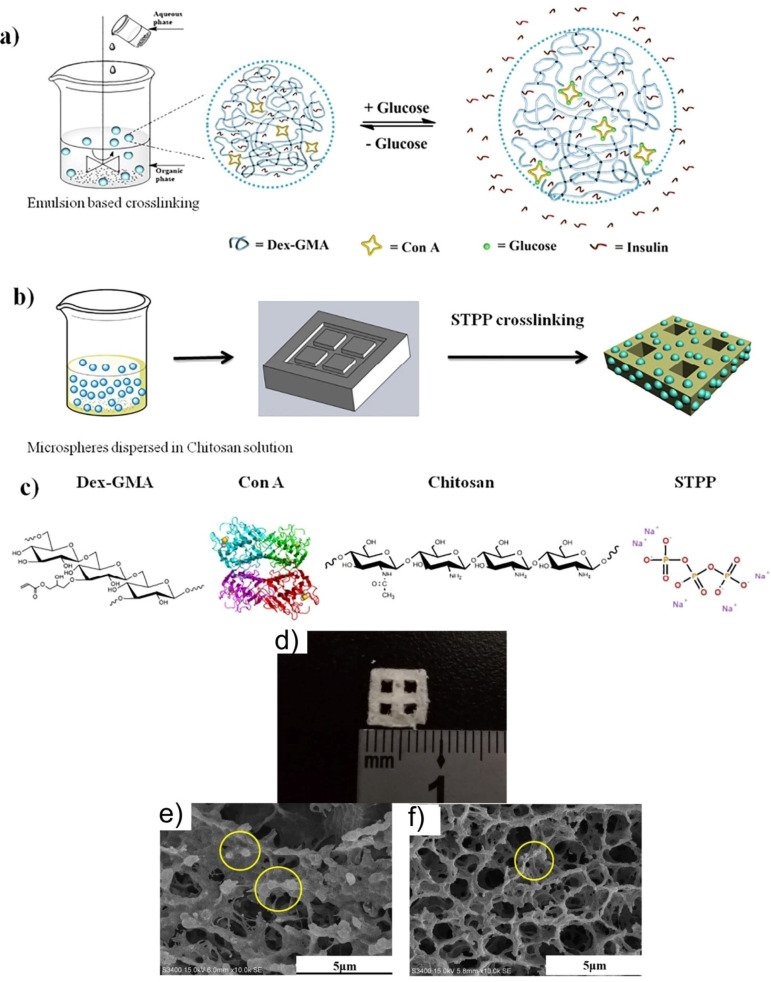
A glucose-sensitive platform: schematic representation of (**a**) synthesis of glucose-responsive microspheres; (**b**) scaffold preparation. (**c**) The chemical structure of system components. SEM images of (**d**) surface, (**e**) cross-section, and (**f**) scaffold containing insulin-loaded microspheres. Reproduced from [105], with permission from © 2018 Elsevier.

For production of a glucose-sensitive platform, different systems used the lectin special characteristics of carbohydrate binding. The most used lectin in insulin-driven drug delivery is concanavalin A (Con A) due to its four binding sites. Yin et al. showed that for an ideal insulin treatment, the ConA micro/nanospheres inserted in a polymer matrix can provide good results (Figure 8) [105]. They obtained a pancreatic system intended to release insulin in a long-term and close-looped manner. The system was engineered by using a chitosan hydrogel whose degradation allowed the leak out of insulin microspheres, enabling an extended insulin delivery.

Liu et al. obtained a polyelectronic nanocomplex (PEC) for insulin delivery by synthesis of chitosan-g-polyethylene glycol monomethyl ether (CS-g-mPEG) copolymers with various mPEG graft concentrations [106]. They studied the hypoglycemic effect by *in vivo* oral administration. The action of two copolymers, a CS-g-mPEG nanocomplex and fabricated mPEG-CS, was compared and the results demonstrated the best absorption was achieved by CS-g-mPEG at a graft ratio of 10%. Another outcome of this work was the implication of nanoparticles containing insulin in the penetration of the mucus of the intestine.

Other authors compared the blood glucose-lowering effect in diabetic rats, in the cases of oral administration versus insulin subcutaneous injection. They used nanocarriers made from calcium carbonate, covered with hyaluronic acid [107]. The delivery of insulin to the small intestine was effectively conducted by an enteric-coated capsule loaded with chitosan-poly(γ-glutamic acid) nanoparticles [108]. The release of insulin was evaluated *in vitro* using carboxymethyl chitosan-phenylboronic acid-Lvaline multifunctional nanoparticles [109].

The production of biodegradable nanomaterials is favorable for targeted drug release applications due to their bioavailability, biocompatibility, better retention time, lower toxicity, and enhanced permeability. A widely utilized protein polymer is gelatin due to its nontoxic and biodegradable properties [118].

#### 2.3.2. Enzyme-Responsive Nanomaterials

Among all stimuli-responsive systems, enzyme-responsive systems are suitable choices in biomedical applications due to their superior selectivity, easy decomposition, mild conditions, and properties such as sensitivity, biorecognition, catalytic efficacy, and process efficiency. In nature, bacteria situated in different organs produce certain enzymes such as hydrolytic (e.g., glycosidases) or reductive (e.g., azoreductase) which can degrade different kinds of polysaccharides, for example, cyclodextrin, pectin, dextrin, and chitosan. In the case of enzyme-responsive polymeric nanoplatforms, enzymes are utilized to break up the polymer to obtain desirable properties [110,119].

**Figure 9:**
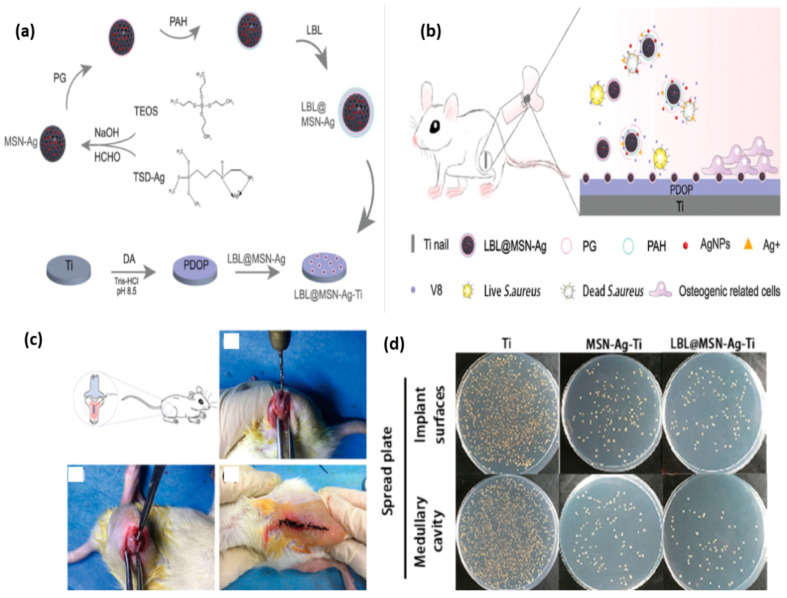
The enzyme-sensitive nanosystem. (**a**) The illustration of the mechanism for obtaining LBL@MSN-Ag-Ti substrates. (**b**) Illustration of infection cure and bone tissue growth. (**c**) The osteogenic response after implantation in infected femurs in rats. (**d**) The antibacterial activity on the different implant surfaces after one week of implantation. Reproduced from [110], with permission from © The Royal Society of Chemistry 2020.

Ding et al. obtained an enzyme-sensitive nanoplatform (Figure 9) intended to cure infections associated with *S. aureus* and facilitating the growth of bone tissue *in vivo* [110]. They reported the fabrication of a titanium-based implant containing mesoporous silica nanoparticles (MSNs) loaded with silver nanoparticles (Ag NPs) coated with multilayer layers of poly(L-glutamic acid) (PG) and polyallylamine hydrochloride (PAH) (LBL@MSN-Ag modified Ti substrates). They used a model of a bacterium-infected femur from a rat.

Enzyme-responsive drug delivery systems are usually built from nanomaterials such as polymers or inorganic materials. Different enzymes particular to certain tumors can influence the peptide structure or the ester bonds from nanocarriers, by releasing the loaded drug at desired locations [120,121,122]. In enzyme-responsive drug delivery systems, the most used triggers are proteases and phospholipases [122]. Proteases are especially favorable in manufacturing these systems since they are usually overexpressed through inflammation, cancer, and infection. Trypsin, as a fundamental stomach-related proteinase, commands exocrine secretion of the pancreas, which is connected with the incitement of a few more stomach enzymes [123,124]. Phospholipase A2 (sPLA2) is picking up a lot of consideration in the therapeutic field because of its upregulation in the tumor microclimate. For example, a few authors demonstrated the efficiency of the phospholipase-responsive liposome (PSL) in drug delivery, due to the presence of (sPLA2) in the tumor cells triggering liposome degradation. sPLA2 is liable in initiating and conducting the peptide nucleic acid PNA release [125].

Other authors engineered an enzyme-responsive nanoplatform for the theranostics of breast cancer by using self-assembled nanoparticles of an N-(2-hydroxypropyl methyl) acrylamide copolymer-gadolinium- paclitaxel-Cyanine5.5 conjugate. The enzymatic trigger was the tetrapeptide GFLG which facilitated the disruption of the high-molecular weight conjugate in low-molecular weight cathepsin B products, releasing the anticancer drug in the tumor [126]. Adenosine triphosphate-coated (ATC) enzyme-sensitive silver nanoparticles are especially engaging because of their astounding stability in a typical physiological climate and controllable suppression of liver carcinoma cells in humans [111]. New insights for developing an intelligent drug release nanoplatform for controllable glioblastoma therapy were described. Activatable low-molecular weight protamine—poly(ethylene glycol)-poly(ε-caprolactone)—nanoparticles (ALMWP-NP) demonstrated remarkably superior protease-dependent glioma controllable reactions [112]. The layer-by-layer technique was utilized for improving the design of carrier systems. A nanoplatform formed from poly(2-oxazoline)-based materials was engineered to initiate thrombolysis via a urokinase plasminogen activator [113].

### 2.4. Dual and Multi-Responsive Nanomaterials

A step forward for biomedical applications is attained when the smart nanomaterials are simultaneously sensitive to more stimuli. The nanomaterials which are sensitive to a few sorts of stimuli are the key for expanding the efficacy of drug delivery and for supporting the diagnosis by monitoring a few physiological changes at once.

The development of nanomaterials with both diagnostic and therapeutic properties is the most powerful technological frontier for moving forward to nanotheranostics. The demand for these technologies is based on the advantage of multiple functions such as multimodal imaging, synergistic therapies, and targeting. The working system of nanotheranostics depends on biological, chemical, and physical triggers considering the activation of the diagnostic and/or the therapeutic properties only at the infected site. In this era of the “war on cancer”, the dual and multi-stimuli-responsive methodology is undeniably appropriate for theranostics as some properties can provide diagnostics, while others could initiate therapy and curing. Consequently, multi-stimuli-sensitive polymers are drawing in expanding consideration for their advantages in the biomedical field.

**Figure 10:**
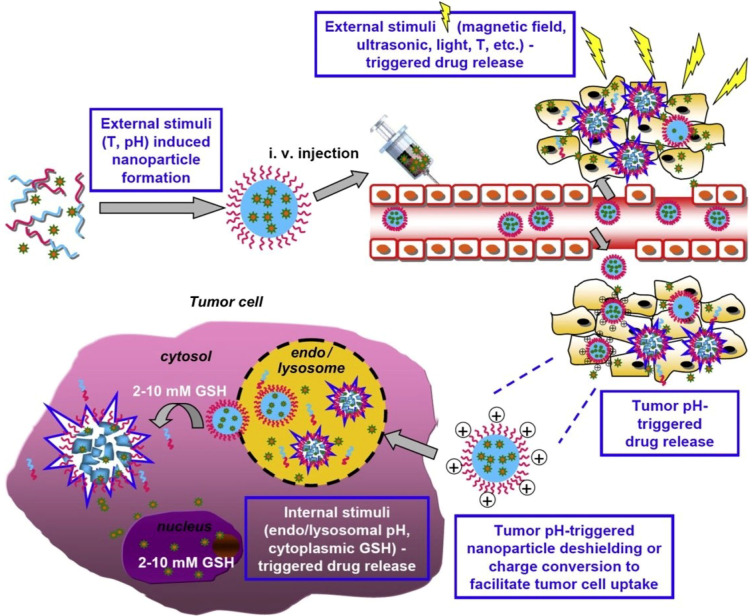
Scheme of internal and external multi-stimuli action in the case of a polymeric nanoparticle material. Reproduced from [127], with permission from © 2013 Elsevier.

Multi-stimuli-sensitive polymeric nanoparticles were developed as emerging targeting drug delivery systems. In Figure 10, a scheme of internal and external multi-stimuli action is outlined [127]. External stimuli such as temperature and pH facilitate the emergence of nanoparticles, while stimuli such as light, the magnetic field, the temperature, and ultrasonic are intended to control drug delivery. Examples of multi-sensitive nanomaterials and their applications are listed in Table 4.

In the actual global fight against cancer, the development of new diagnosis and treatment methods is crucial. The research community developed different systems from multi-responsive stimuli nanomaterials which are proven to be effective in cancer treatment. Yu et al. obtained by a distillation precipitation polymerization a multi-responsive system with controllable drug release and tumor cells destruction. The pH-, redox-, and temperature-responsive drug release system realizes the penetration and the accumulation of the tumor and targeted drug delivery [128].

A novel system responsive to both pH and ultrasound stimuli was obtained by Chen and Du, based on a poly(ethylene oxide, 2-(diethylamino)ethyl methacrylate, (2-tetrahydrofuranyloxy)ethyl methacrylate PEO-*b*-P(DEA-*stat*-TMA) block copolymer [129]. The anticancer drugs were encapsulated in the system and then released in a controllable way after the action of ultrasound radiation or varying pH stimuli. Figure 10 highlights the obtaining of the pH- and ultrasound-sensitive PEO_43_-*b*-P(DEA_33_-*stat*-TMA_47_) vesicle. Ultrasound radiation and the solution pH influence the system, leading to a smaller vesicle. The system disruption and re-self-assembly are due to the ultrasound radiation.

A lab-on-a-chip based on a magnetic- and temperature-responsive nanoparticle system was developed to capture diagnostic targets [130]. The supermagnetic nanoparticle has γ-Fe_2_O_3_ as a core, surrounded by self-assembled carboxylate-terminated PNIPAAm as the temperature-responsive agent (with LCST). Biotin and streptavidin were loaded in this nanoparticle and the final device has walls from PEG-modified polydimethylsiloxane channels. Above MCST, the system formed magnetic-responsive aggregates and a further increase in temperature combined with the magnetic field application induced immobilizations of these aggregates on the microchannel wall. If the temperature is decreased below the LCST and the magnetic field is removed, the aggregates are redispersed and eluted through the channel. Those nanoparticles are useful for capturing diagnostic targets at the right time and at a certain channel position.

A recent study describes a novel dual stimuli-sensitive system for a controllable tumor drug delivery. A chemo-photothermal synergetic cancer therapy was achieved by integrating DNA aptamer with dopamine-reduced graphene oxide (rGO-PDA) nanosheets [131]. The rGO-PDA nanosheets acted at once as an NIR photothermal agent, inducing hyperthermia for photothermal therapy.

Some authors obtained a dual stimuli response nanoplatform with superior targeting capacity, good photothermal conversion properties, and smart drug release sensitive to both pH and photothermal heating. The nanosystem was effectively utilized to release DOX to protein tyrosine kinase 7 (PTK7)-overexpressing cancer cells in a controllable manner, by reacting simultaneously to NIR irradiation and to the acidic intracellular environment. The mix of a dual nanocarrier of drug loading and dual stimuli release is effective in chemo-photothermal synergetic therapy.

A new approach of a pH- and temperature-sensitive mixed ferrite nanohybrid was recently obtained for theranostic applications [132]. The system was obtained from polyethyleneimine cross-linked Pluronic F127. Rhodamine isothiocyanate (RITC) and folic acid (FA) were tethered to the nanoparticles which were then entrapped with DOX to produce (DOX-FA-Poly-MFNPs) with a thermo/pH-efficient drug delivery pattern and therapeutic activity.

Temperature and pH stimuli-responsive microcapsules for controlled drug release applications were obtained by Chen et al. [133]. Dual stimuli-responsive pNIPAM particles were obtained and loaded with oil-soluble fluorescent green (OG) and Nile red (NR). The pH stimulus just set off the release of the OG molecule yet did not have any effect on the NR molecules inside the particles. The NR molecules inside the particles were released in the case of higher temperatures, due to the collapse of microcapsules, but the OG molecules did not.

The multidisciplinary behavior of CuS NPs, very useful for theranostics applications through different methods, renders it appropriate for building up a novel system for cancer management [134]. Those nanoplatforms that can be used for treatment (phototherapy and combinatorial therapy) and visualization (photoacoustic imaging and magnetic resonance imaging) have also been discussed. Zhang et al. obtained a dual sensitive system, with both pH and reduction responsiveness, for improved photodynamic and photothermal therapy [135]. Thiol-modified polylysine (PLL) nanoparticles with disulfide bonds were modified with poly(ethylene glycol) (PEG). The singlet oxygen generation and thermoresponsiveness of the loaded ICG in dimethylmaleic anhydride DMMA-modified PLL were efficient in destroying cancer cells.

**Figure 11:**
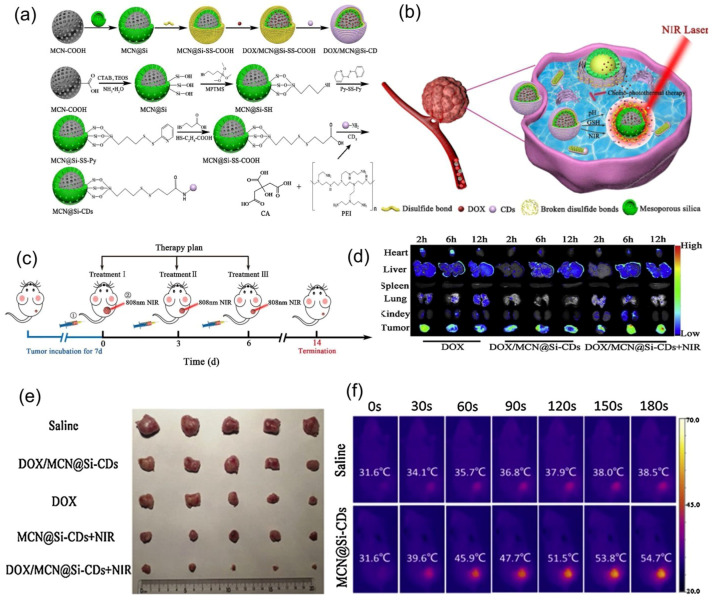
A nanoplatform made of a silica-coated carbon nanocomposite with redox/NIR/pH stimuli-responsive release ability and superior chemo-photothermal combined antitumor treatment activity. (**a**) Schematic illustration of synthesis of DOX/MCN@Si-CDs and MCN@Si-CDs. (**b**) Schematic illustration of chemo-photothermal combined antitumor treatment. (**c**) Schematic therapy plan for *in vivo* treatments: ① intravenous injection in rat tail; ② NIR tumor irradiation. (**d**) Organs and tumor fluorescence images. (**e**) Excised tumors images. (**f**) Mice thermal imaging photographs. Reproduced from [136], with permission from © 2020 Elsevier.

Figure 11 presents a multi-stimuli-responsive innovative system for controlled drug delivery, and thermal-chemotherapy for tumor treatment was described by Lu et al. [136]. They obtained a novel mesoporous silica-coated carbon nanocomposite (DOX/MCN@Si-CDs) with redox/NIR/pH stimuli-responsive release ability and superior chemo-photothermal combined antitumor treatment activity as compared with individual treatments.

As cancer is still one of the most complex diseases which need pragmatic multi-step procedures, the significance of customized medication, focused on a more individualized treatment, has motivated the examination of nano-based conducted diagnosis and therapeutics (theranostics). As the expectation is to “take out two targets with one shot”, researchers have just depicted the arising idea to engineer “intelligent” nanosystems for simultaneously realizing diagnosis and therapy. These nanosystems used in fighting against cancer are based on nanoplatforms which could efficiently co-entrap photosensitizers and chemotherapy, dendrimers, functional nanocarriers, drug-loaded nan-aggregates, liposomes, hybrid nanoparticles, and nanogels for “on-demand” anticancer drug delivery [137,138,139,140,141,142,143,144,145,146,147,148,149]. All of these systems are composed of multi-stimuli-responsive versatile materials, which enable them to ensure the expected requirements. The intrinsic properties of these materials, such as enzyme overexpression, low pH, huge redox potential, high concentration of reactive oxygen species, and high temperature in a tumoral environment, provide controllable drug delivery. Since those features may not be sufficient in all cases to trigger the nanotheranostic abilities of the system, some additional stimuli such as ultrasound, an electric or magnetic field, and light are used to provide the expected results with little side effects but high efficiency.

A theranostic approach was developed by Li et al., by obtaining a simple glutathione-responsive turn-on nanoparticle (DHP). For DHP synthesis, the authors used a disulfide bond-linked hydroxyethyl starch paclitaxel conjugate (HES-SS-PTX) and an NIR dye, Dioctadecyl-3,3,3,3-tetramethylindotricarbocyanine iodide (DiR). They compared the photothermal conversion efficiency of the DHP prepared as described and the free DiR and obtained values of 23.5% and 6.9%, respectively, after an irradiation with an 808 nm laser. They demonstrated the dual modal imaging (fluorescent and photoacoustic) *in vivo* capability and antitumor activity through chemo-photothermal synergistic therapy of the dual stimuli-responsive system [150].

## 3. Advances in Plasmonic Nanomaterials

A special class of smart nanomaterials is derived from plasmonic nanoparticles used in innovative sensitive tools for diagnostics and therapeutics. The collective electronic (plasmon) resonances of noble/coinage metal nanoparticles enable a strong optical response essential in applications such as photocatalysis, sensing, photothermal heating, and enhanced fluorescence. Biomedical applications rely on plasmonic nanoparticles’ properties to absorb or scatter light at near-infrared wavelengths, transmissive in the human body [151]. A large number of applications misuse the extraordinary properties of metals to support electromagnetic waves at their surfaces, through the oscillation of their conduction electrons known as surface plasmons. The local dielectric environment, size, structure, shape, and composition determine the surface plasmon polariton modes enabling nanostructures to focus and direct light down to the nanoscale. The ability of plasmonic nanostructures to strongly interact with light at wavelengths that significantly exceed their dimensions led to the appearance of the nanoplasmoic field [152]. Consequently, most recent strategies for the design and manufacture of plasmonic nanostructures for accurately controlling light have opened new entryways for the applications that were recently perceived as impossible.

Recent studies have shown that by targeting gold nanoparticles to the cell nucleus region, the nuclear stiffness is enhanced, slowing down the migration and invasion speed of cancer cells and suppressing metastasis [153]. Further, gold nanoparticles exhibit high contrast in photothermal therapeutic treatments, as well as photoacoustic, optical coherence, and X-ray CT imaging. Conjugates of gold nanoparticles present augmented binding affinity, long circulatory half-life, size-enhanced tumor uptake, increased targeting selectivity, high biocompatibility, and rapid transport kinetics. If all those properties are put together in a highly multifunctional platform, one can obtain an increasingly selective and potent oncologic treatment [154].

As diagnosis is the key in the screening and treatment of human diseases, modern-day researchers developed sensitive tools for real-time and accurate tracking of the treatment effect. In a recent paper, the authors obtained a core–shell structure MPs@ SiO2@Pd–Au with a crystalline magnetic core, amorphous silica interaction layer, and Pd–Au shell for medulloblastoma diagnosis and radiotherapy evaluation. Owing to the plasmonic and alloying effects, MPs@SiO2@Pd–Au may contribute to efficient electron transfer and high surface stability under laser irradiation during the laser desorption/ionization process [155]. Other authors described the design of a plasmonic gold nano-island (pGold) chip assay for enhanced diagnosis and monitoring of myocardial infarction [156]. A multifunctional platinum nanoreactor intended for point-of-care metabolic analysis, visual detection, and mass spectrometry fingerprinting for *in vitro* pancreatic cancer diagnostics was designed using controlled core–shell structured Fe3O4@SiO2@Pt particles [157]. Another work describing the application of laser desorption/ionization mass spectrometry in large-scale clinical *in vitro* cervical cancer diagnosis utilized a plasmonic chip with Au nanoparticles deposited on a dopamine bubble layer [158].

An increasing interest was paid to the field of thermoplasmonics, defined as plasmonic nanoparticles remotely controlled by light to release heat on the nanoscale volumes. The capability of using plasmonic materials as photothermal agents is based on a combination of properties such as the high density of free electrons, the absence of thermobleaching, resonances that enhance light–matter interaction, and low losses for noble metals. Those materials are best choices in applications requiring spatially confined heating, such as in nanosurgery and photoacoustic and photothermal imaging [159]. Recent works described the capability of gold nanorods to convert NIR radiation into heat for antibacterial application without affecting cells’ viability and proliferation [160] and of keratin-coated gold nanoparticles to kill the brain cancer cells by photothermal therapy [161]. The photothermal therapy induced by the presence of gold nanoparticles in a system capable to develop immunotherapy represents a major breakthrough in the fight against malignant solid tumors. This synergistic new approach was comprehensively described moving from *in vivo* studies to clinical trial applications in patients suffering from solid tumors. Although those systems hold great promise in nanomedicine, there are still risks involved, such as the wrong cells being targeted, unknown long-term side effects, and unwanted immune reaction systems. For this reason, the combination of hyperthermia with chemotherapeutic activity or cancer immunotherapy demonstrated improved care of oncological patients [162].

The properties of Au and Ag nanoparticles have inspired the field of plasmonic nanoparticles in the last two decades, but recently, non-noble metals have been the subject of quickly expanding interest as less expensive, more practical alternatives. Colloidal nanocrystals functionalized with silica have been utilized for plasmon-driven photocatalysis and surface-enhanced Raman spectroscopy at visible and near-infrared wavelengths due to their enhanced stability in water and efficient broadband photothermal heating [163].

## 4. Conclusions

This mini-review highlights the tremendous progress of nanotechnology over the last two decades and why it is of vital importance for the further study of smart materials for biomedical applications. The manufacture of stimulus-responsive nanomaterials involves a creative formulation of drugs and polymers so that they respond to biological cues such as differences in pH and temperature between healthy and diseased tissue and the induced response leads to controlled and sustained release of the load. Better understanding of the physiological changes and of the differences between healthy and diseased tissue will improve the possibility of designing materials that uniquely respond to local stimuli. The synthesis of nanoparticles for drug release after encapsulation must be tailored as a function of the therapeutic goals.

The efficacy of diagnosis and therapeutics is achieved by designing multifunctional nanoplatforms to provide new methods and strategies for future clinical oncology nanomedicine. Nanotheranostic improvement maybe speaks to the most elevated level of innovative development in the nanomedicine field. Another important field in nanomedicine is lab-on-a-chip innovation, which includes the scaling down of processes that occur on an integrated platform. Stimuli-responsive nanopolymers on the lab-on-a-chip systems have contributed essentially to the decrease in cost, time, efficient reagents, and tests required. Some complex biological *in vitro* barriers such as improving the biocompatibility, stability, and biodegradability; ensuring nontoxic, timed turns on and off; and precise control of response sites of stimuli-responsive nanoplatforms still remain difficult to realize. In spite of these hurdles, knowledge about plasmonic nanoparticles offers great possibilities for enhanced diagnosis and monitoring for prognostic use towards point-of-care testing. However, even if the macroscopic photothermal effects such as fluid convection, tissue damage, drug release, or chemical reactions were intensively studied, the actual temperature of the plasmonic nanoparticles during these processes was often unknown. In that sense, the recent developments in efficient thermal imaging techniques are expected to contribute to further insight. The synergistic new approach moving from *in vivo* studies to clinical trial applications in the oncological field holds great promise in nanomedicine but must be studied in more depth.

In brief, with the increasing progress and the continuous innovation of science and technology, we have reason to believe that stimuli-responsive nanomaterials will surely lead to effective strategies and bring a substantial benefit in the biomedical field.

## Figures and Tables

**Table 1:** Examples of physico-responsive nanomaterials and their biomedical applications.

Nr. Crt.	Stimuli	Nanomaterial	Application	Reference
1.	Temperature	*Poly(ethylene oxide)_a_-poly(propylene oxide)_b_-* *poly(ethylene oxide)_a_* PEO-PPO-PEO	Oral drug delivery, wound healing	[24]
2.	Temperature	*Gold nanoparticles—Pluronic^®^F127-Hydroxypropyl methylcellulose* AuNPs-PF127-HPMC	Drug delivery, photothermal platform, skin wound healing	[25]
3.	Temperature	*Poly(oligo(ethylene glycol) methacrylate –co-poly(glycidal methacrylate) copolymers/poly(lactic* *acid-co-glycolic acid)* P(OEGMA-co-PGMA) copolymers/PLGA	Tissue engineering	[26]
4.	Temperature	*Collagen- or chitosan-based*	Drug delivery	[27]
5.	Temperature	*Poly(N-isopropylacrylamide)- poly(N,N-dimethylacrylamide)- poly(acrylic acid)* PNIPAM-PDMA-PAA	Drug delivery	[28]
6.	Temperature	*Poly(Nisopropylacrylamide-co-sulfobetaine methacrylate) nanogel* PNS nanogels	Diagnosis/chemotherapy	[29]
7.	Electrical	*Poly(3,4-ethylenedioxythiophene)-coated poly(lactic acid-co-glycolic acid) nanofiber* PEDOT-coated PLGA nanofiber	Drug delivery	[30]
8.	Electrical	*Fe_3_O_4_/Polyaniline* Fe_3_O_4_/PANI	Antimicrobial, drug delivery	[31]
9.	Electrical	*Polyaniline/gold nanocomposite* PANI/AuNCs	Immunosensor detection of chronic kidney disease	[32]
10.	Electrical	*Polyaniline, poly(3,4-ethylenedioxythiophene)* PANIP, PEDOT	Neural prostheses	[33,34]
11.	Electrochemical	*Biosynthesized gold nanoparticles/ poly(catechol)/graphene sheets/glassy carbon electrode* Bio AuNP/Pol/Gr/GCE	Biosensor, DNA mutation and acute lymphoblastic leukemia detection	[35]
12.	Light	*poly(ethylene glycol)* PEG	Switchable fluorescent probes	[36]
13.	Light	*Ruthenium-containing block copolymer* Poly-Ru nanoparticles	*In vivo* photodynamic therapy and photochemotherapy	[37]
14.	Magnetic	*Fe_3_O_4_/methoxy poly(ethylene glycol)-poly- (lactide) composite nanocapsules* Fe_3_O_4_/MePEG-PLA composite nanocapsules	MRI	[38]
15.	Magnetic	*Trastuzumab (Tra, a humanized monoclonal antibody that specifically recognizes HER2)- doxorubicin* *poly(vinyl alcohol)/ single-component thiol-functionalized poly (methacrylic acid) T-DOX* PVA/PMASH magnetic nanocapsules	Tumor therapy	[39]
16.	Magnetic	*3D collagen hydrogel*	Directed neuronal regeneration	[40]

**Table 2:** Examples of chemical-responsive nanomaterials and their biomedical applications.

Nr. Crt.	Stimuli	Nanomaterial	Application	Reference.
1.	pH	*Ppoly (ethylene glycol)-Ag nanoparticle* PEG-Ag NPs	Antibacterial, wound healing	[77]
2.	pH	*Hybrid ultra-pH-sensitive (HyUPS) nanotransistor* HyUPS nanotransistors	Receptor-mediated endocytosis in tumor cells	[78]
3.	pH	*Layered double hydroxides-zinc (II) phthalocyanine containing octasulfonate nanohybrid* LDH-ZnPcS_8_ nanohybrid	Theranostics	[79]
4.	pH	Melanin-like nanoparticles	Photoacoustic imaging of tumors	[80]
5.	pH	*polylactic acid-Resveratrol* PLA-RSV	Drug delivery	[81]
6.	pH	*Poly(carboxybetaine methacrylate)-nanodiamonds* PCBSA-@-NDs	Theranostics	[82]
7.	Redox	*Poly (ethylene glycol)-Pluronic F68-nanoscale covalent organic frameworks* F68@SS-COFs	Cancer therapy	[83]
8.	Redox	*Hyaluronic acid–chitosan–lipoic acid nanoparticles* (HACSLA-NPs)	Breast cancer therapy	[84]
9.	Redox	*Folate redox-responsive chitosan nanoparticles* FTC-NPs	Anticancer drug delivery	[85]
10.	Redox	*Poly (ethylene glycol) conjugated to paclitaxel* via *disulfide linkage* PEG_2000_-S-S-PTX	Prodrug for breast cancer cells	[86]
11.	Redox	*Prodrug/AgNPs hybrid nanoparticles*	Drug delivery	[87]
12.	Redox	*P[(2-((2- ((camptothecin)-oxy)ethyl)disulfanyl)ethylmethacrylate) -co- (2-(D-galactose)methylmethacryl-ate)] and silver nanoparticles* P(MACPTS-co-MAGP)@AgNPs nanoparticles	Drug release	[88]

**Table 3:** Examples of biological-responsive nanomaterials and their biomedical applications.

Nr. Crt.	Stimuli	Nanomaterial	Application	Reference.
1.	Glucose	*Acetalated dextran nanoparticles* Ac-Dex Nps	Glycemic control	[103]
2.	Glucose	*Boronic acid-derived polymers*	Drug delivery	[104]
3.	Glucose	*Glycidyl methacrylated dextran/Concanavalin A* Dex-GMA/Con A ConA micro/nanospheres	Insulin treatment	[105]
4.	Glucose	*Chitosan-g-polyethylene glycol monomethyl ether nanocomplex* CS-g-(mPEG) NP	Oral insulin delivery	[106]
5.	Glucose	*Hyaluronic Acid (HA)-coated calcium carbonate NPs*	Oral insulin delivery	[107]
6.	Glucose	Chitosan/poly(gamma-glutamic acid) nanoparticles	Oral insulin delivery	[108]
7.	Glucose	*Carboxymethyl chitosan-phenylboronic acid-Lvaline nanoparticles* (CMCS-PBA-LV) NPs	Oral administration of insulin	[109]
8.	Enzyme	*Nanoplatform formed from Ti substrates modified with* *layer-by layer mesoporous silica nanoparticles-silver nanoparticles* LBL@MSN-Ag nanoparticles	Tissue growth *in vivo* and, simultaneously, treat implant-associated bacterial infection	[110]
9.	Enzyme	*Adenosine triphosphate coated with silver nanoparticles* ATP-Ag nanoparticles	Participate in signal transduction and protein activity	[111]
10.	Enzyme	*Activatable low-molecular weight protamine—poly(ethylene glycol) poly(ε-caprolactone) nanoparticles—loaded with paclitaxel* ALMWP-NP-PTX	Glioblastoma therapy	[112]
11.	Enzyme	*Layer-by-layer assembly of poly(2-oxazoline)-based materials*	Therapeutic delivery	[113]

**Table 4:** Examples of dual and multi-responsive nanomaterials and their biomedical applications.

Nr. Crt.	Stimuli	Nanomaterial	Application	Ref.
1.	pH/redox/temperature	*N,N0 -bis(acryloyl)cystamine, Poly(N-isopropylacrylamide), 2-hydroxyethylmethacrylate, Methacrylic acid, a disulfide bond contained cross-linker, and doxorubicin* SS-NPs@DOX	Drug delivery	[128]
2.	Ultrasound/pH	*Poly(ethylene oxide, 2-(diethylamino)ethyl methacrylate, (2-tetrahydrofuranyloxy)ethyl methacrylate* PEO_43_-*b*-P(DEA_33_-*stat*-TMA_47_)	Drug release	[129]
3.	Temperature/magnetic field	*Poly(N-isopropylacrylamide)- Magnetic nanoparticles* b-PNIPAM-mNPs	The isolation of diagnostic targets that can be used in point-of-care devices	[130]
4.	Light/pH	rGO-PDA nanosheets	Drug delivery, phototherapy	[131]
5.	pH/magnetic field	*Magnetic nanoparticles* MFNPs	Targeting, drug delivery, MRI	[132]
6.	Temperature/pH	*Poly(N-isopropylacrylamide)* pNIPAM	Drug release	[133]
7.	pH/light/enzyme	*Copper sulfide nanoparticles* CuS NPs	Theranostics	[134]
8.	pH/redox	*Thiol-modified polylysine- indocyanine green/ poly(ethylene glycol) nanoparticles* PLL-ICG/DPEG Nps	Photothermal and photodynamic therapy	[135]
9.	pH/redox	*Poly (ethylene glycol) –polylacticacid-thioketal groups-Paclitaxel-(Maleimide thioether) Chlorin e6* mPEG-PLA-TKI-PTX nanoparticles and Ce6-(SS-mal-)-Ce6 (PNPCe6)	Chemotherapy, drug release	[136]
10.	pH/redox	*Histidine -4 polyamidoamine dendrimer -Disulfide bonds- (poly (ethylene glycol)- Transferrin* (His-PAMAM-ss-PEG-Tf, HP-ss-PEG-Tf) nanocarrier	Anticancer drug delivery	[137]
11.	pH/redox	*Lipoic acid ethylenediamine- Polyethylene glycol diglycidyl ether- _L_lysine* poly(LAE-co-PGDE-co-Lys) core-crosslinked nano aggregate	Anticancer drug delivery	[138]
12.	pH/redox	*Paclitaxel- poly(6-O-methacryloyl-d-galactopyranose)- gemcitabine/ N-acetyl-d-glucosamine(NAG)-poly(styrene-alt-maleic anhydride)-b-polystyrene* PTXL-ss-PMAGP-GEM/NAG NLCs	Anticancer drug delivery	[139]
13.	UV light/redox/pH	*Six-arm star-shaped amphiphilic copolymer with poly (caprolactone) -bpoly (acrylic acid) -b-poly (poly (ethylene glycol) methyl ether methacrylate)*	Anticancer drug delivery	[140]
14.	pH/temperature	Poly(NIPAM)nanogel @ Fe_3_O_4_ NPs/poly(acrylic acid) -graft—κ—carrageenan	Drug delivery	[141]
15.	Redox/pH/temperature	*Nanogels based on alginate and cystamine*	Anticancer drug delivery	[142]
